# The diagnostic value of a non-contrast computed tomography scan-based radiomics model for acute aortic dissection

**DOI:** 10.1097/MD.0000000000026212

**Published:** 2021-06-04

**Authors:** Zewang Zhou, Jinquan Yang, Shuntao Wang, Weihao Li, Lei Xie, Yifan Li, Changzheng Zhang

**Affiliations:** a Department of Radiology, the First Affiliated Hospital of Guangzhou University of Chinese Medicine; b The First Clinical Medical College of Guangzhou University of Chinese Medicine, Guangzhou, China.

**Keywords:** aortic dissection, multi-slice computed tomography, radiomics, receiver operating characteristic curve

## Abstract

To investigate the diagnostic value of a computed tomography (CT) scan-based radiomics model for acute aortic dissection.

For the dissection group, we retrospectively selected 50 patients clinically diagnosed with acute aortic dissection between October 2018 and November 2019, for whom non-contrast CT and CT angiography images were available. Fifty individuals with available non-contrast CT and CT angiography images for other causes were selected for inclusion in the non-dissection group. Based on the aortic dissection locations on the CT angiography images, we marked the corresponding regions-of-interest on the non-contrast CT images of both groups. We collected 1203 characteristic parameters from these regions by extracting radiomics features. Subsequently, we used a random number table to include 70 individuals in the training group and 30 in the validation group. Finally, we used the Lasso regression for dimension reduction and predictive model construction. The diagnostic performance of the model was evaluated by a receiver operating characteristic (ROC) curve.

Fourteen characteristic parameters with non-zero coefficients were selected after dimension reduction. The accuracy, sensitivity, specificity, and area under the ROC curve of the prediction model for the training group were 94.3% (66/70), 91.2% (31/34), 97.2% (35/36), and 0.988 (95% confidence interval [CI]: 0.970–0.998), respectively. The respective values for the validation group were 90.0% (27/30), 94.1% (16/17), 84.6% (11/13), and 0.952 (95% CI: 0.883–0.986).

Our non-contrast CT scan-based radiomics model accurately facilitated acute aortic dissection diagnosis.

**Key points**

- Early diagnosis of acute aortic dissection (AAD) based on clinical signs is challenging.
- Non-contrast-enhanced CT imaging is convenient, but its sensitivity for AAD diagnosis is insufficient.
- We extracted radiomics features from non-contrast-enhanced CT images to construct a model that facilitated AAD diagnosis.

## 1 Introduction

Acute aortic dissection (AAD) is a pathological change in the aorta, characterized by the formation of true and false lumens in local regions. Tears in the aortic intimal layer allow blood from the aortic lumen to enter the intima-media space, forcing these layers to separate along the principal aortic axis direction. Many factors might cause aortic dissection, the most frequent of which are hypertension and atherosclerosis.^[1]^ AAD incidence is approximately 2.9 to 3.5 per 100,000 persons, with a significant increasing trend in recent years.^[2]^ AAD mortality rate can reach 50% if the patients are not treated within 48 hours.^[3]^ Therefore, early and accurate AAD diagnosis is crucial.

Previous studies have shown that less than half of AAD patients presented typical AAD clinical signs during physical examination,^[4]^ resulting in delayed diagnosis or even misdiagnosis.^[5]^ Currently, AAD diagnosis depends primarily on contrast-enhanced CT angiography.^[2]^ However, many patients cannot be examined by contrast-enhanced CT angiography due to their clinical condition or allergy to the contrast agents. Non-contrast CT is more convenient and can provide a faster diagnosis than contrast-enhanced CT angiography. However, it is less sensitive for differentiating between the AAD-associated true and false lumens than contrast-enhanced CT angiography. Ultrasonography is currently the preferred screening method for AAD, but it also has some limitations, including higher requirements of the doctors’ diagnostic experience and operating techniques.^[6]^

Currently, radiomics receive the attention of an increasing number of radiology scholars for its potential ability to help detect lesions, improve diagnostic accuracy, predict disease risk, and guide treatment strategies.^[7]^ To make use of its advantages and provide a reference for clinical diagnosis, we developed a radiomics model based on non-contrast CT images and evaluated its efficacy in diagnosing AAD, an undertaking never reported before.

## 2 Materials and methods

### 2.1 Patient data

The study was approved by the Ethics Committee of the First Affiliated Hospital of Guangzhou University of Chinese Medicine, and the batch number is NO.ZYYECK (2019) 157. We performed a retrospective analysis of AAD patients diagnosed based on different diagnostic criteria at the First Affiliated Hospital of Guangzhou University of Chinese Medicine from October 2018 to November 2019. The institutional review board approved the study, and the need for informed consent was waived. All patients assigned to the dissection group satisfied the following criteria: had clinically diagnosed AAD; non-contrast CT and CT angiography images were available; and were not treated by interventional therapy or surgery before the CT examination. Patients satisfying the following criteria were assigned to the non-dissection group: admission to the hospital due to chest pain; and AAD and other vascular lesions not detected on non-contrast CT and CT angiography. Patients were excluded from both groups if at least one of the following conditions was met: clinical or image/video data were insufficient; and the image could not be used for analysis due to poor quality. We retrospectively selected 100 individuals for this study using these criteria (50 for each group). The dissection group comprised 42 male and 8 female patients, whose ages ranged from 25 to 92 years (mean ± standard deviation [SD], 56.7 ± 15.3 years). The non-dissection group comprised 40 male and 10 female patients, whose ages ranged from 24 to 80 years (mean ± SD, 54.5 ± 12.7 years). These 100 patients were divided into a training group (70 patients) and a validation group (30 patients) by the random number table method.

### 2.2 CT image collection

The CT images were acquired by Discovery CT 750 HD (GE Healthcare, Chicago, IL). The average scanning range of the non-contrast CT extended from the upper part of the aortic arch to the vascular bifurcation. CT scanning was performed with the following settings: field of view, 350 × 350 mm; tube voltage, 120 kV; tube current, 35 mAs; rotation speed, 0.6 s; pitch, 0.984; slice thickness, 1.25 mm. Contrast-enhanced scanning was performed after injecting 1 mL/kg body weight of a contrast agent with a high-pressure syringe at an injection rate of 1.5 mL/s, followed by injection of 30 mL normal saline at the same injection rate.

### 2.3 Image analysis

#### 2.3.1 Depicting the region of interest

**Figure 1:**
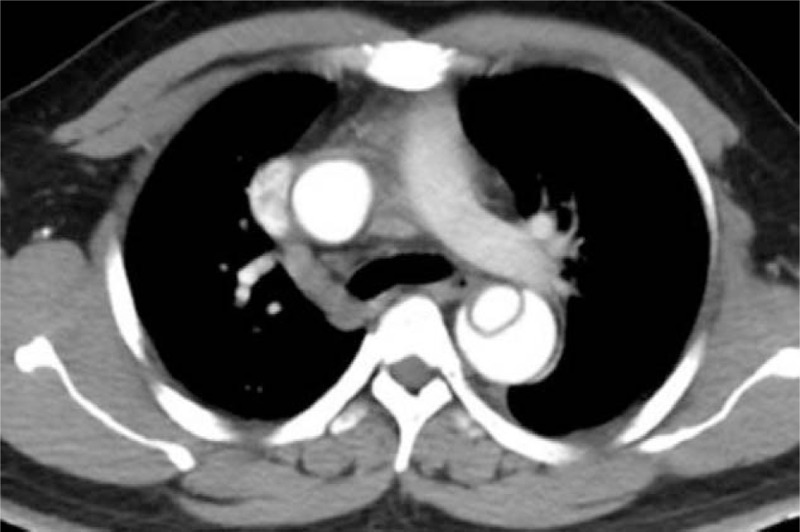
A 31-year-old male patient. The true and false lumens are revealed in these contrast-enhanced CT angiography images. CT = computed tomography.

**Figure 2:**
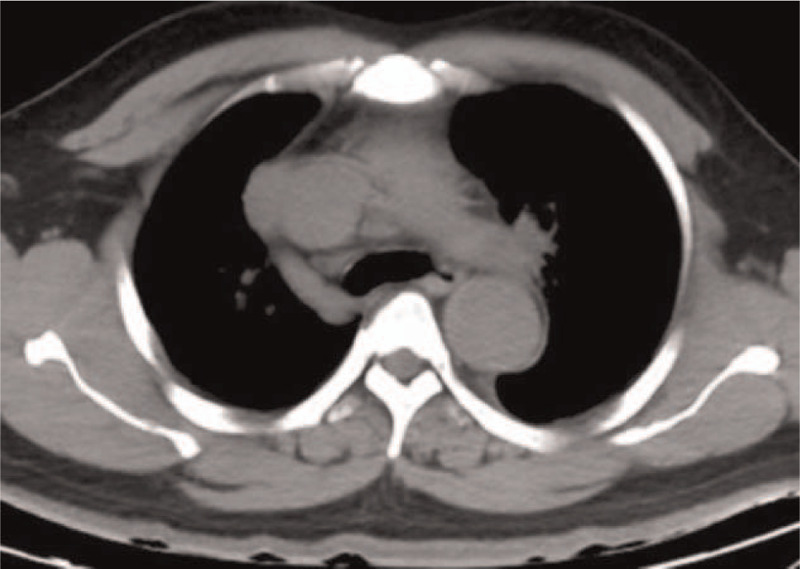
No apparent abnormality is observed at the corresponding layer in the non-enhanced CT image of the same patient as in Fig. 1. CT = computed tomography.

**Figure 3:**
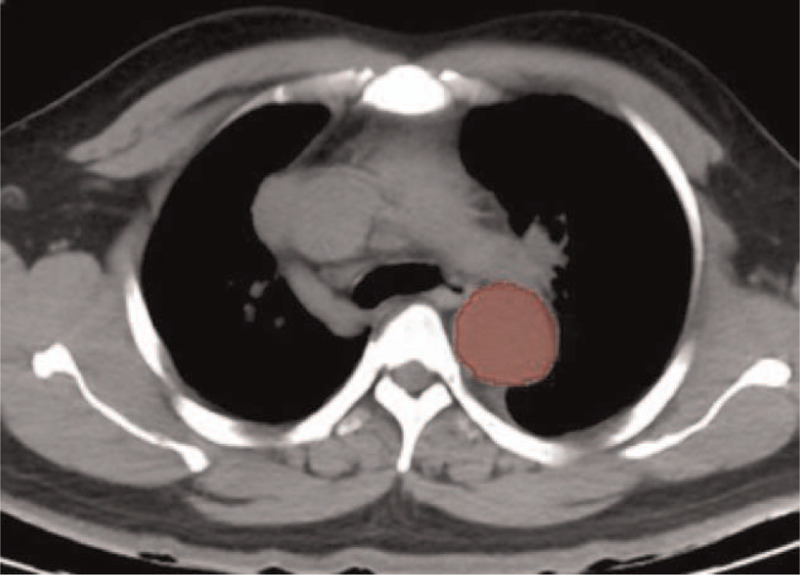
A schematic of the region-of-interest (ROI) depicted in this layer.

CT images were imported into the 3D Slicer software (https://www.slicer.org) and processed by a radiologist with 3 years of CT diagnosis experience. Based on the locations of the aortic dissection shown in the CT angiography images, the radiologist marked the corresponding areas in the non-contrast CT images, generating 50 regions-of-interest (ROIs) for each study group. Representative images showing the ROI depiction are presented in Figs. 1–3. One week later, CT images of 30 randomly selected patients were processed by the same radiologist and a second radiologist with 4 years of CT diagnosis experience. Both radiologists depicted the ROIs independently and extracted the characteristic features from the images to evaluate intra- and inter-observer consistency.

#### 2.3.2 Radiomic features extraction

**Figure 4:**
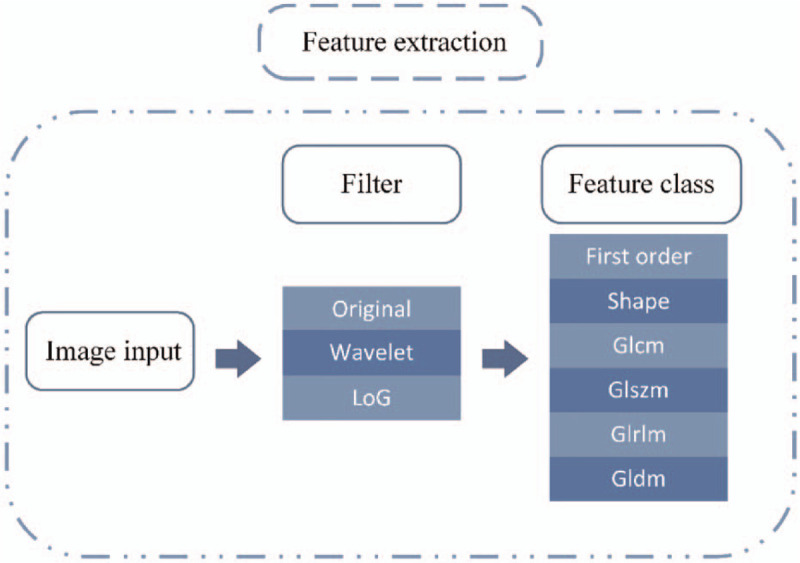
A schematic showing radiomic features extraction by the PyRadiomics platform.

All images were resampled at a voxel size of 1 × 1 × 1 mm. Voxel values were aggregated into 25-HU wide bins to reduce image noise interference and to normalize their intensities. PyRadiomics (http://www.radiomics.io/pyradiomics.html),^[8]^ an open-source package in Python (https://www.python.org/), was used to extract 3 types of characteristics: first-order statistics, geometric descriptive features, and texture features. Texture features included the grey-level co-occurrence matrix, size-zone matrix, run-length matrix, and difference matrix.^[9,10]^Figure 4 shows the feature extraction schematic.

### 2.4 Statistical analysis

The Shapiro-Wilk test and Levene test were used to compare the groups for clinical data (age, aortic diameter). Comparisons of age and aortic diameter within each group were made by *t* test. Pearson chi-squared test analyzed differences in sex, smoking status, hypertension status, and aortic calcification between the groups. Differences in intimal flap clarity in the non-contrast CT images were examined using the Fisher exact test. Differences with a *P*-value <.05 were considered statistically significant.

Consistency of the radiomics features extraction between samples from the same and different groups was also evaluated. We used intra-group and inter-group correlation coefficients (ICC) to evaluate the consistency in the extracted image features from 30 randomly selected images between the first and second extraction sessions by the first radiologist and between the first and second radiologists in the second extraction session.^[11]^ The feature consistency was divided into poor reliability (ICC < 0.5), medium reliability (0.5 ≤ ICC ≤ 0.75), and high reliability (ICC > 0.75).^[12]^

The radiomic features were first extracted from the CT images on the PyRadiomics platform. Subsequently, the Lasso regression^[13]^ was used to reduce the dimensions of characteristic parameters with ICC > 0.75. Next, characteristic parameters with non-zero coefficients were selected to construct radiomic tags. Finally, logistic regression analysis was used to perform a bi-classification prediction of the radiomic tags and construct the non-contrast CT scan-based radiomics model. The radiomics model diagnostic performance was evaluated by a receiver operating characteristic (ROC) curve.

Two different radiologists (both with over ≥3 years of CT diagnostic experience) were blinded to the clinical and imaging diagnoses and scored the 100 patients based on non-contrast-enhanced CT images only. Scoring was on a scale from 1 to 5 (1, normal; 2, probably normal; 3, uncertain; 4, probably aortic dissection; 5, aortic dissection). Based on their aortic dissection scores, the radiologists’ diagnostic performance was evaluated by assessing the area under the ROC curve (AUC).

**Figure 5:**
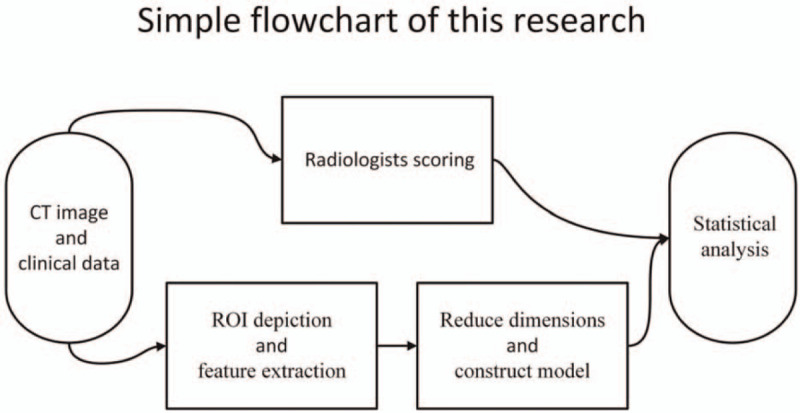
A flow chart showing the study design.

Statistical analysis was performed using R (Version 3.5.2, https://www.r-project.org/). The ICC results were processed by the “psych” package, Lasso regression by the “glmnet” package, and ROC analysis by the “pROC” package. A flowchart of this study is shown in Fig. 5.

## 3 Results

### 3.1 Clinical data

The 2 patient groups differed in hypertension, clarity of intimal flap in the non-contrast CT images, and aortic diameter. The groups were similar in age, sex, smoking status, and aortic calcification (Table 1). A comparison between the training and validation groups is shown in Table 2.

**Table 1:** Clinical data of the dissection group and non-dissection group.

Characteristic	Dissection group (n = 50)	Non-dissection group (n = 50)	*t*/*χ*^2^	*P*
Age	56.7 ± 15.3	54.5 ± 12.7	–0.767	.445
Sex				
Male	42	40	0.271	.603
Female	8	10		
Hypertension				
Yes	36	12	23.077	<.001
No	14	38		
Smoking				
Yes	18	16	0.178	.673
No	32	34		
Dekabey classification				
Dekabey I	16	/	/	/
Dekabey II	3	/	/	/
Dekabey III	31	/	/	/
Intimal flap				
Clear	29	0	/	<.001
Blurred	16	0	/	<.001
None	5	50	/	<.001
Aortic calcification				
AAO	13	6	0.793	.373
AOA	26	20	1.449	.229
DAO	24	17	2.026	.155
Aortic diameter, mm				
AAO	40.8 ± 7.5	30.6 ± 5.2	7.930	<.001
AOA	36.9 ± 9.2	25.5 ± 3.5	–8.194	<.001
DAO	34.6 ± 9.6	24.3 ± 3.7	–7.121	<.001

**Table 2:** Clinical data of the training and validation groups.

Characteristic	Training group (n = 70)	Validation group (n = 30)	*t*/*χ*^2^	*P*
Age	55.6 ± 12.6	55.7 ± 17.2	−0.047	.963
Sex				
Male	58	24	0.116	.733
Female	12	6		
Hypertension				
Yes	41	7	10.447	.001
No	29	23		
Smoking				
Yes	24	10	0.008	.927
No	46	20		
Dekabey classification				
Dekabey I	11	5	0.014	.905
Dekabey II	3	0	/	.552
Dekabey III	19	12	1.623	.203
Intimal flap				
Clear	17	12	2.519	.113
Blurred	11	5	0.014	.905
None	42	13	2.357	.125
Aortic calcification				
AAO	14	5	0.152	.697
AOA	31	15	0.276	.599
DAO	30	11	0.333	.564
Aortic diameter, mm				
AAO	35.3 ± 8.5	36.6 ± 7.5	−0.723	.473
AOA	30.8 ± 8.2	32.1 ± 10.7	−0.603	.550
DAO	34.6 ± 9.6	24.3 ± 3.7	−0.493	.625

### 3.2 Inter- and intra-group consistency in the extracted radiomic features

We extracted 1203 radiomic features using the PyRadiomics platform. The imaging features extracted by the first radiologist were highly consistent between the 2 trials (ICC = 0.873–0.994). There was also high consistency in the extracted imaging features between the two radiologists (ICC = 0.763–0.960). The results described below are therefore based on the features extracted by the first physician during the first trial.

### 3.3 Construction of the radiomics model

**Figure 6:**
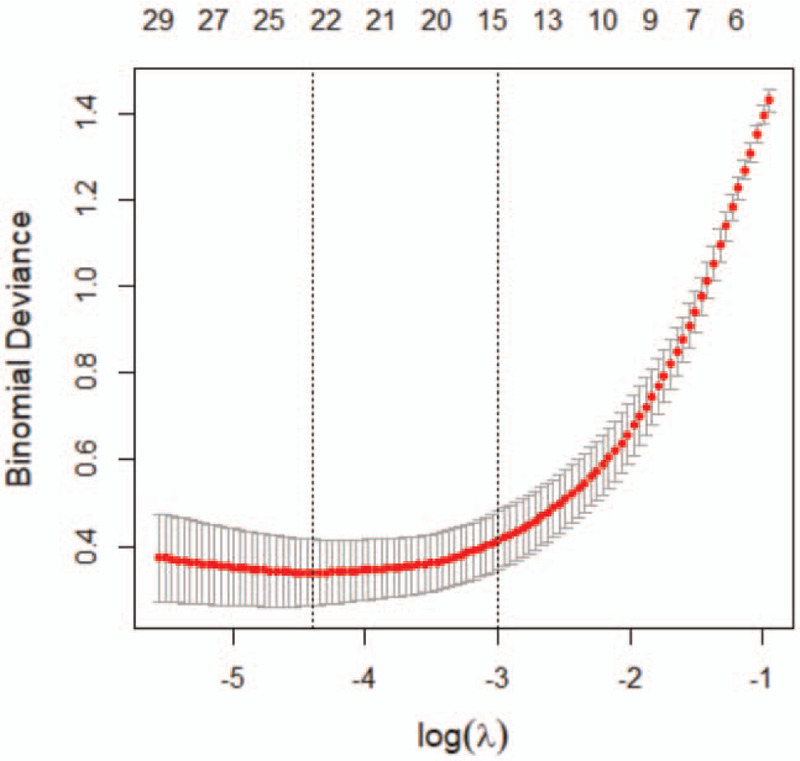
Leave-one-out cross-validation in Lasso regression yielded the binomial deviation of the model and its relationship with the regularization parameter log(*λ*). The dotted line on the left represents the radiomics model when using an optimum *λ* of 0.030718. The dotted line on the right represents the radiomics model using the smallest coefficient and *λ* within one standard deviation from its optimum value (the corresponding value of *λ* is 0.077883).

**Figure 7:**
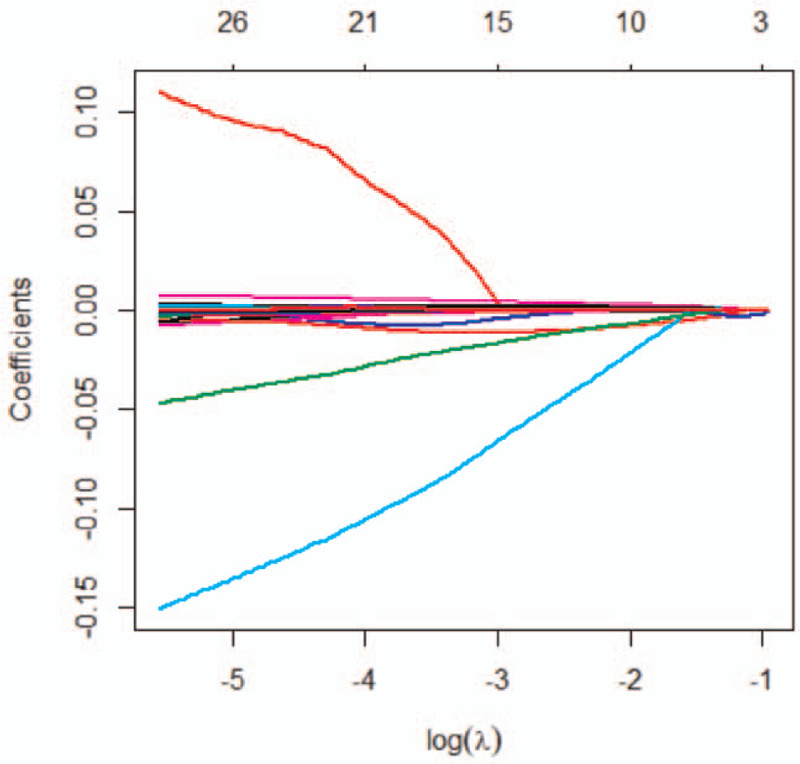
The relationship between the feature coefficient and log(*λ*) in Lasso regression. This figure describes the converging process of the feature coefficients. We obtained 14 characteristic features with non-zero coefficients when *λ* = 0.077883.

Fourteen features with non-zero coefficients were selected from the 1203 radiomic features using Lasso regression (Figs. 6 and 7). The coefficient of the constant term was 13.05569. The linear combination of the selected features, obtained by multiplying them with the corresponding coefficients and then adding the constant term, were used as radiomic tags (Equation 1). Finally, logistic regression was used to construct the radiomics model for AAD diagnosis based on non-contrast CT images.

*Rad_score* = 13.05569

+ (3.765755 × log-sigma-1-0-mm-3D_glcm_JointEntropy)

−(0.002155 × log-sigma-1-0-mm-3D_glrlm_LongRunEmphasis)

−(0.000025 × log-sigma-2-0-mm-3D_fristorder_Energy)

−(0.004111 × log-sigma-3-0-mm-3D_ fristorder_Range)

−(0.000060 × log-sigma-3-0-mm-3D_glrlm_GrayLevelNonUniformity)

+(0.003138 × log-sigma-4-0-mm-3D_glrlm _LongRunEmphasis)

−(0.012173 × log-sigma-5-0-mm-3D_glcm_DifferenceVariance)

−(0.014171 × wavelet-HLL_fristorder_Kurtosis)

+(0.000233×wavelet-LLH _gldm_GrayLevelNonUniformity)

+(0.001100×wavelet-HLH_gldm_SmallDependenceEmphasis)

−(0.016766×wavelet-HHH_firstorder_Kurtosis)

+(0.000077×wavelet-HHL_firstorder_TotalEnergy)

+(0.004168×wavelet-LLL_gldm_Imc1)

+(0.000362×original_firstorder_SmallDependenceEmphasis) (Equation 1).

### 3.4 Identification and prediction performances of the radiomics model and physician's scores

**Figure 8:**
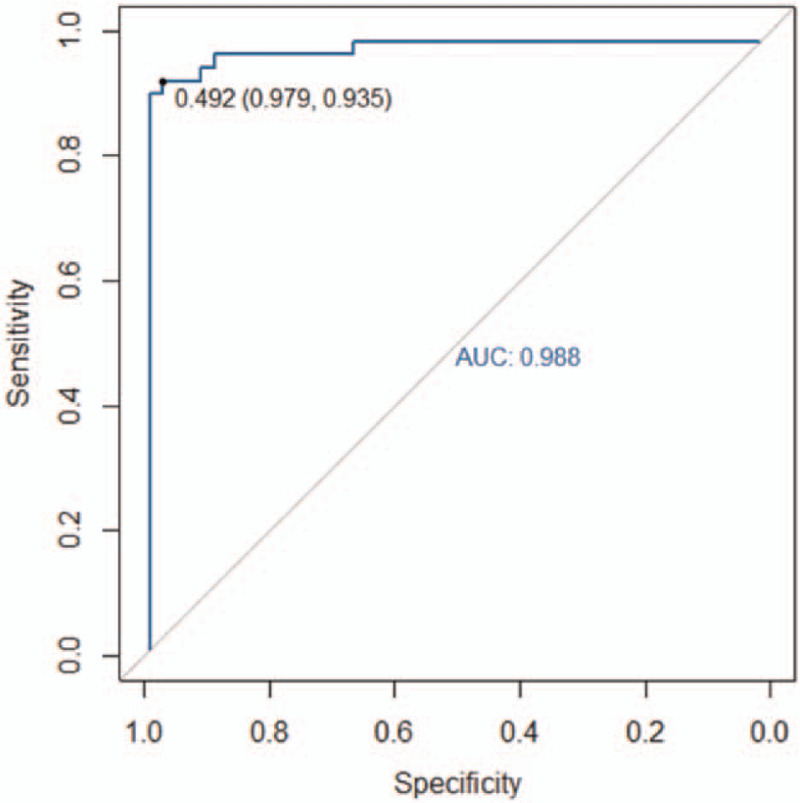
The area under the receiver operating characteristic (ROC) curve (AUC) of the non-contrast CT scan-based radiomics model for the training group was 0.988. CT = computed tomography.

**Figure 9:**
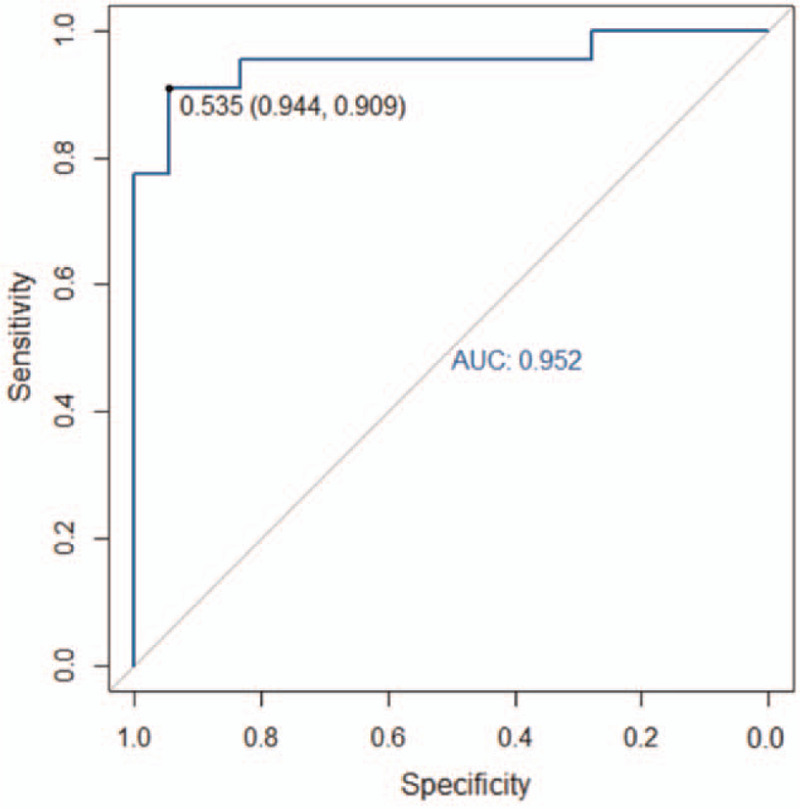
The area under the receiver operating characteristic (ROC) curve (AUC) of the non-contrast CT scan-based radiomics model for the validation group was 0.952. CT = computed tomography.

The accuracy, sensitivity, specificity, and AUC of the radiomics model for the training group were 94.3% (66/70), 91.2% (31/34), 97.2% (35/36), and 0.988 (95% CI: 0.970–0.998), respectively. The respective values for the validation group were 90.0% (27/30), 94.1% (16/17), 84.6% (11/13), and 0.952 (95% CI: 0.883–0.986). The ROC curves of the training and validation groups are shown in Figs. 8 and 9, respectively.

The AUCs obtained by the 2 radiologists based on the non-contrast CT images alone were 0.879 (95% CI: 0.810–0.948) and 0.894 (95% CI: 0.831–0.957), respectively.

## 4 Discussion

The concept of radiomics was first proposed by Lambin et al^[14]^ in 2012. Using a high-throughput feature extraction algorithm, radiomics allows the extraction of many quantitative features by data mining. Such an approach represents a breakthrough over the traditional image diagnosis based on morphology. It also provides a quantitative tool for accurate diagnosis based on medical imaging. To date, radiomics has been applied to many different fields, including molecular typing of tumors,^[15,16]^ staging classification,^[15,17]^ differential diagnosis,^[18]^ selection of treatment options,^[19]^ efficacy testing,^[20]^ and prognosis evaluation.^[21,22]^ Non-contrast CT scanning sensitivity for differentiating between AAD-associated true and false lumens is still insufficient. A clear intimal flap was visible on non-contrast CT images in only 29 of the 50 patients in the dissection group. Furthermore, the traditional imaging diagnostic accuracy was greatly affected by subjective factors such as the physician experience and fatigue level. Therefore, a transition from morphology-based semi-quantitative imaging diagnosis to accurate quantitative diagnosis would be an important future trend in medical imaging. This study's contribution to this trend is the development of a non-contrast CT scan-based radiomics model for diagnosing AAD.

### 4.1 Analysis of patient clinical data

Hypertension is an independent risk factor for AAD.^[23,24]^ In this study, the hypertension status differed significantly between the dissection and non-dissection groups. A study by Januzzi et al^[25]^ showed that >67% of AAD patients were diagnosed with early-stage hypertension. The dissection group was mostly comprised of men (42/50 patients). The groups also differed in the diameter of the ascending aorta, aortic arch, and descending aorta. AAD patients usually have a larger aortic diameter than non-AAD individuals. Of the dissection group patients, 31 were classified as Dekabey III, 16 as Dekabey I, and only 3 as Dekabey II. This result indicates that the tear in the aorta of most AAD patients starts from the distal opening of the clavicular artery. The groups were similar in the calcification states of the ascending aorta, aortic arch, and descending aorta. Thus, aortic calcification had little diagnostic value for AAD. While there was a significant difference between the groups in intimal flap occurrence in the non-contrast CT images, the non-contrast CT images of a significant number of AAD patients showed no or a very blurred depiction of an intimal flap. This finding suggests that AAD diagnosis based on the appearance of an intimal flap on non-contrast CT images has some limitations. From a clinical perspective, a more accurate method is required to diagnose AAD based on non-contrast CT images.

### 4.2 Radiomics analysis

In this study, we constructed a radiomics model based on non-contrast CT images. This model exhibited good performance and high accuracy. The high-throughput feature extraction algorithm used in radiomics essentially allows image digitalization. Radiologists require the image and, more importantly, the patients’ data.^[26]^ These data are often difficult to identify by the naked eye. In this study, the AUCs of AAD diagnosis based on non-contrast CT images, performed by 2 radiologists with over 3 years of CT diagnosis experience, were 0.879 and 0.894, respectively. These values were lower than the validation group AUC of 0.952. This difference suggests that the non-contrast CT scan-based radiomics model performed better at diagnosing AAD than the 2 radiologists when only non-contrast CT scan images were available. In terms of efficiency, it takes several minutes for a radiologist to diagnose AAD in a patient. In contrast, the radiomics model could evaluate the risk of AAD for hundreds of patients in just a few seconds.

The non-invasive nature of the non-contrast CT scan-based radiomics model for AAD makes it more advantageous and convenient than biological marker diagnostic approaches such as D-dimer diagnosis. Asha and Miers^[27]^ reported that D-dimer concentration, an indicator of fibrinolytic function, could be used to diagnose AAD. When using a concentration of 500 ng/mL as cut-off value, the diagnostic sensitivity and specificity of D-dimer were 95.7% and 61.3%, respectively. The hierarchical model used by Watanabe et al^[28]^ to evaluate D-dimer diagnostic performance for AAD yielded an AUC of 0.950. The diagnostic performances of the models developed by Asha and Miers^[27]^ and Watanabe et al^[28]^ were both inferior to the non-contrast CT scan-based radiomics model developed in this study. This finding suggests that our radiomics model can achieve a more precise diagnosis of AAD.

The radiomics model, comprised of 6 first-order statistical features and 8 texture features, had very high sensitivity and specificity for AAD diagnosis. Furthermore, the model demonstrated very high accuracy in the training and validation groups. These results indicated that the proposed algorithm had small variance and deviation, making it suitable for solving the AAD diagnosis problem. Besides, the use of Lasso regression to reduce radiomics feature dimensions presented a leave-one-out cross-validation for the proposed method. Such validation improves the robustness of our radiomics model.

### 4.3 Limitations

This study had some limitations. Only the largest lesion layer was selected for depicting the ROI; thus, the ROI failed to cover all the layers in the lesion. Fifty patients were included in each group, making the number of patients analyzed in this study relatively small. In the future, we intend to depict a ROI covering all layers in the lesion and increase the number of samples as part of a multicenter study.

## 5 Conclusion

In summary, the non-contrast CT scan-based radiomics model developed here could be of great value for AAD diagnosis.

## Author contributions

**Conceptualization:** Zewang Zhou, Jinquan Yang, Shuntao Wang, Lei Xie, Yifan Li, Changzheng Zhang.

**Data curation:** Yifan Li.

**Formal analysis:** Zewang Zhou, Yifan Li.

**Investigation:** Zewang Zhou, Lei Xie.

**Methodology:** Zewang Zhou, Jinquan Yang, Shuntao Wang, Weihao Li, Lei Xie.

**Project administration:** Zewang Zhou.

**Software:** Zewang Zhou, Weihao Li.

**Supervision:** Zewang Zhou.

**Visualization:** Zewang Zhou.

**Writing – original draft:** Zewang Zhou, Shuntao Wang.

**Writing – review & editing:** Zewang Zhou, Changzheng Zhang.
